# How Do Small Adjustments in Apical Surface Area Impact Tissue-Wide Homeostasis?

**DOI:** 10.17912/micropub.biology.001727

**Published:** 2025-08-06

**Authors:** Mohammadreza Hosseini Moghadam, Hadiya Omer, Anastasia Dilello, Lia Campbell-Enns, Paula Pineda, Derek Neufeld, Sofia Ertulova, Stephen Ingram, Ernest Ho, Tim Rogalsky, Juan Nicolas Malagon

**Affiliations:** 1 Birmingham City University, Birmingham, England, United Kingdom; 2 Grant Park High School, Winnipeg, Manitoba, Canada; 3 University of Manitoba, Winnipeg, Manitoba, Canada; 4 Canadian Mennonite University, Winnipeg, Manitoba, Canada

## Abstract

Epithelial tissues, the primary origin of most cancers, undergo complex changes in organization and size. Understanding epithelial mechanisms is crucial for elucidating how their disruption leads to cancer, despite the stochastic nature of their dynamics. This study investigates complex spatial and temporal patterns of apical cell area (ACA) oscillations during sex comb rotation in the male *Drosophila melanogaster* forelegs, using *ImageJ* . We discovered that, although ACA oscillations appear irregular, the degree of size variation is consistent and predictable. These findings suggest such irregular oscillations may contribute to fine-tuning mechanisms and play a role in maintaining epithelial homeostasis.

**Figure 1. Fruit fly leg as a model for studying irregular cell oscillations:**
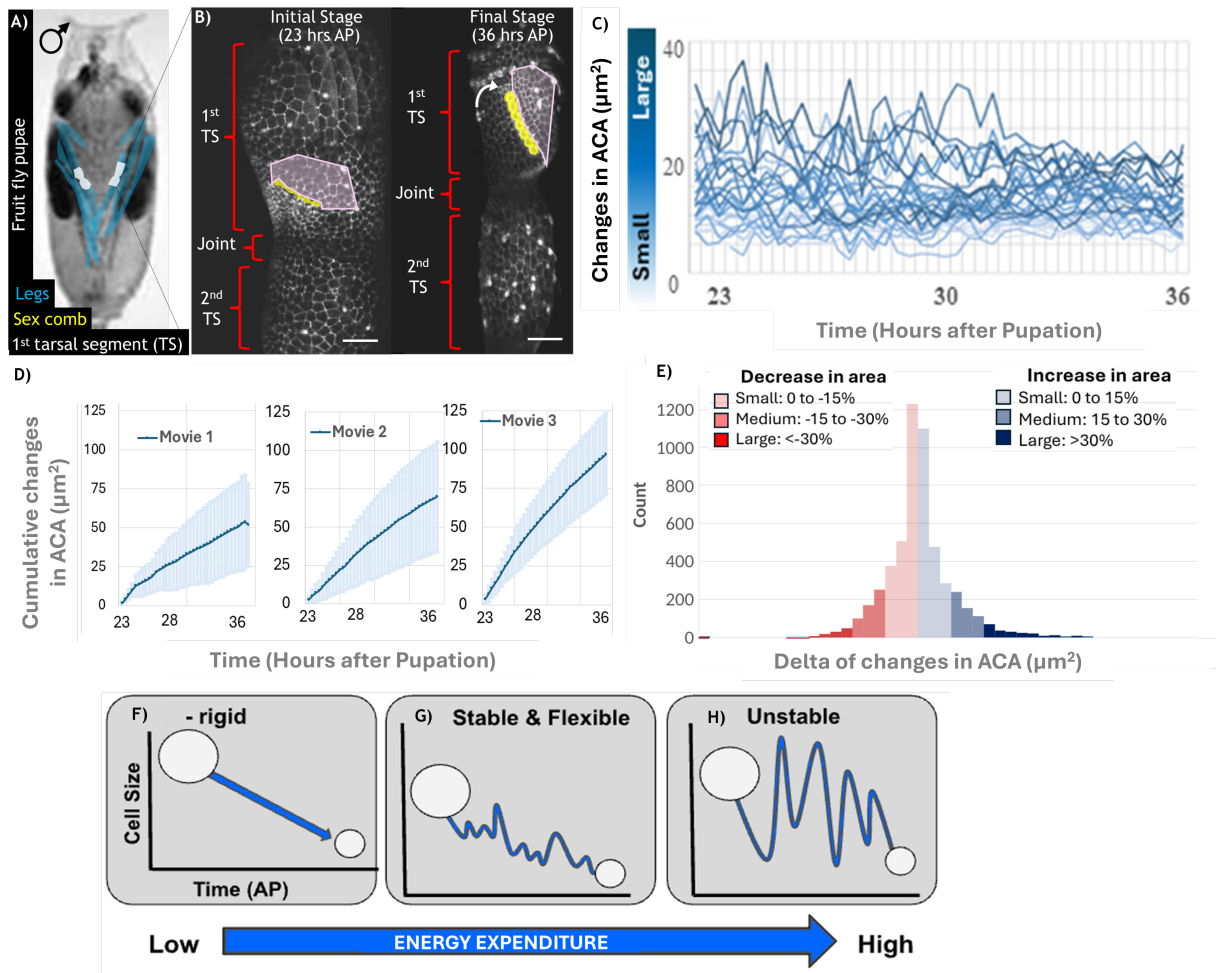
**(A)** Fruit fly pupa. **(B)** Confocal image of a developing foreleg. In the developing pupal legs (shaded blue in A), the 1st and 2nd tarsal segments (shaded white) highlight distinct regions of cell crowding. The 1st and 2nd tarsal segments are labelled as TS1 and TS2, respectively, with the sex comb marked in yellow. Scale bar: 20 µm. The cells above the comb (shaded pink) display a reduction in tissue area via irregular oscillations. **(C)** Diagram showing temporal changes in apical cell area (ACA), with irregular oscillations that appear unpredictable. **(D)** Cumulative Changes in Apical Cell Area (ACA) During Development. The dark blue trendline represents the average cumulative changes, with error bars showing the standard deviation. Cumulative changes exceeded 15% by a significant margin, with average cumulative changes of 50%, 75%, and 90% in Mov. 1, Mov. 2, and Mov. 3, respectively. **(E)** Distribution of Fluctuations in Apical Area. A total of 5,332 area changes were recorded across all cells between each slice. As shown by the histogram, small changes represented 74.5%, medium changes accounted for 19.2%, and large changes comprised 6.2%. This indicates that the majority of changes were approximately 1 µm². **(F-H)** Models proposed for how to arrange tissue proximal to the sex comb. Subtle oscillations identified in the results align with model **(G)** .

## Description

Epithelia are sheets of interconnected cells that form fundamental tissue structures essential for animal morphogenesis (Harris and Tepass, 2010). Understanding epithelial development is particularly important, as most cancers originate from epithelial tissues (Méndez-López, 2022). In *Drosophila* , like other insects, epithelium consists of a single layer of cells, unlike the stratified, multilayered structure of vertebrate skin (Tepass et al., 2001). This structural simplicity makes *Drosophila* a powerful model for studying epithelial biology. In recent years, research on epithelial morphogenesis has revealed that changes in tissue size often involve oscillations in ACA (David et al., 2010; Ho et al., 2018; Martin, 2010). Our previous work has shown that the epithelium proximal to the sex comb in *Drosophila* legs serves as a useful model for studying the role of apical cell oscillations in tissue remodeling (Ho et al., 2018).

Sex comb rotation in *D. melanogaster* provides an excellent model for investigating the dynamics of epithelial changes in tissue size (Doerksen et al., 2022). The sex comb consists of a linear row of specialized bristles located on the forelegs of male flies (Atallah, 2008; Kopp, 2011). During pupal development, this structure undergoes a characteristic rotation—from a position perpendicular to the leg's long axis to one that is parallel (Atallah et al., 2009; Ho et al., 2018) ( Fig. 1A- B). Previous studies have shown that, during this process, epithelial cells adjacent to the sex comb undergo active remodeling, including cell extrusion, neighbor exchange, and fluctuations in apical cell area (Ho et al., 2018). Both cell extrusion and neighbor exchange appear to follow unpredictable spatial and temporal patterns, consistent with observations in other systems such as *Drosophila* (Malagón, 2013; Marinari et al., 2012). Additionally, during the time-course study, no cell division occurs in pupal legs (Atallah et al., 2009). This absence of mitosis allows for the tracking of cell shape changes and rearrangements without the confounding effects of cell division—a notable advantage over many vertebrate systems.

Despite rapid advances in the field of epithelial development, it remains unclear how epithelia coordinate such rearrangements while preserving their integrity as a physical barrier (Mira-Osuna and Le Borgne, 2024). Our earlier findings showed that this epithelium experiences a reduction in apical cell area during morphogenesis (Ho et al., 2018). At the cellular level, this reduction is driven by irregular oscillations in apical area (Doerksen et al., 2022). While these fluctuations appear to lack a consistent spatial or temporal pattern ( Fig. 1C ), the present study seeks to explore whether more subtle or emergent patterns underlie this apparent irregularity.

In this study, we show that these irregular oscillations appear to be highly stochastic, yet the magnitude of cell fluctuations exhibits predictable, minor variations in apical cell size. The characteristics of these oscillations—particularly their subtle variability—may support tissue remodeling by enabling a mechanism of precise modulation, while preserving the integrity of the epithelial sheet as a continuous barrier.

This work builds upon our previous study by investigating two additional aspects of the irregular oscillations: (1) the distinction between net and cumulative changes in apical cell area (ACA), and (2) the delta of ACA changes (ΔACA). Our earlier findings showed that comparing initial and final apical cell sizes during sex comb rotation revealed an average net reduction of approximately 15% per cell (Doerksen et al., 2022). However, this net change obscures the dynamic nature of the process, which involves irregular, bidirectional oscillations in cell size. We hypothesized that the cumulative magnitude of these fluctuations would be substantially greater than the net reduction. Quantitative analysis confirmed that the summed magnitude of size changes was significantly larger than the net decrease. In the region proximal to the sex comb, individual cells exhibited cumulative size changes ranging from 50% to 90% ( Fig. 1D ). These findings indicate that cells undergo repeated size adjustments not captured by simple before-and-after comparisons. Notably, we observed no consistent spatial or temporal patterns that could predict the occurrence of these large fluctuations, raising the question of whether these size changes are stochastic.

To investigate the apparent stochasticity of ACA oscillations, we asked whether the magnitude of these changes also lacks discernible patterns. To address this, we quantified the ΔACA, changes between successive time points and found that the distribution of these fluctuations includes both increases and decreases in apical area. These changes fall into three broad categories based on magnitude: (1) large (>30%), (2) intermediate (15–30%), and (3) small (<15%) ( Fig. 1E ). Despite the broad range of fluctuations observed, the vast majority of individual events were small. Approximately 75% of all changes involved minor adjustments in apical area. This suggests that the tissue relies on frequent, fine-scale modifications rather than infrequent, large-scale restructuring.

Our findings show that although the net reduction in apical area is modest, the cumulative magnitude of size changes driven by oscillatory behavior is much higher, potentially imposing a greater energetic burden ( Fig. 1F- H). Nonetheless, most changes are subtle, and large shifts are rare and unpredictable. We suggest that cell extrusion, cell exchange, and these frequent, small-scale oscillations collectively facilitate precise tissue-level rearrangements. This coordinated mechanism likely minimizes energy expenditure, thereby maintaining epithelial integrity during morphogenesis.

These findings align with the concept of emergent properties, highlighting the stratified determinism of biological process, in which seemingly chaotic local behaviors result in coordinated tissue level outcomes.

## Methods

Three time-lapse videos of wild-type male *Drosophila melanogaster* were recorded using confocal microscopy and analyzed according to the protocol previously described (Atallah et al., 2009). For live imaging, pupae were mounted in halocarbon oil (Series 700; Halocarbon Products) on a Sigma coverslip and imaged using a ZEISS LSM 510 laser scanning confocal microscope at 25 °C. A 40× objective lens was used, and imaging was performed with LSM Browser software (ZEISS). Z-stacks were acquired with 3 μm intervals (Atallah et al., 2009).

The videos were analyzed using *ImageJ* software (NIH, http://rsb.info.nih.gov/ij ). 2D projections were created, and epithelial cells near the sex comb were manually outlined and tracked between 23 and 36 hours after pupation (AP). Over 40 cells were labeled in each video (Movie 1: 44 cells, Movie 2: 51 cells, Movie 3: 55 cells), depending on the ease of accurate labeling during morphogenesis. Time points where cells were indistinct were excluded from analysis. Cells lost due to tissue remodeling were also omitted. These cells account for approximately 15–20% of the total cell population in the proximal region. Because they require a distinct analytical approach, they were omitted to avoid introducing bias into our current dataset. Changes in apical cell area were quantified using the “Analyze Particles” tool in ImageJ. Error bars represent ±1 standard deviation.

**Delta of ACA**

The ΔACA was calculated as the difference in apical cell area between two consecutive time points.

**Cumulative changes in ACA**

The cumulative changes in ACA were calculated by summing the absolute value ΔACA over time. The variations in ACA were calculated using the standard deviations.

## Reagents

**Fly strains**

The ubi- *D* Ecad::GFP lines were generated by Oda and Tsukita (2000), and I obtained copies from the Ulrich Tepass laboratory. Flies were reared on a yeast-cornmeal-molasses medium at 25°C.
